# Anti-Alphaviral Alkaloids: Focus on Some Isoquinolines, Indoles and Quinolizidines

**DOI:** 10.3390/molecules27165080

**Published:** 2022-08-10

**Authors:** Anne-Laure Sandenon Seteyen, Emmanuelle Girard-Valenciennes, Axelle Septembre-Malaterre, Philippe Gasque, Pascale Guiraud, Jimmy Sélambarom

**Affiliations:** 1 Unité de Recherche Etudes Pharmaco-Immunologiques (UR-EPI), Université de La Réunion, 97400 Saint-Denis, France; 2 Laboratoire de Chimie et de Biotechnologie des Produits Naturels (CHEMBIOPRO), Université de La Réunion, 97400 Saint-Denis, France; 3 Centre Hospitalier Universitaire de La Réunion, Laboratoire d’Immunologie Clinique et Expérimentale de la Zone Océan Indien (LICE-OI), Pôle de Biologie, 97400 Saint-Denis, France

**Keywords:** alkaloids, arthritogenic alphaviruses, immunomodulators, biological activity

## Abstract

The discovery and the development of safe and efficient therapeutics against arthritogenic alphaviruses (e.g., chikungunya virus) remain a continuous challenge. Alkaloids are structurally diverse and naturally occurring compounds in plants, with a wide range of biological activities including beneficial effects against prominent pathogenic viruses and inflammation. In this short review, we discuss the effects of some alkaloids of three biologically relevant structural classes (isoquinolines, indoles and quinolizidines). Based on various experimental models (viral infections and chronic diseases), we highlight the immunomodulatory effects of these alkaloids. The data established the capacity of these alkaloids to interfere in host antiviral and inflammatory responses through key components (antiviral interferon response, ROS production, inflammatory signaling pathways and pro- and anti-inflammatory cytokines production) also involved in alphavirus infection and resulting inflammation. Thus, these data may provide a convincing perspective of research for the use of alkaloids as immunomodulators against arthritogenic alphavirus infection and induced inflammation.

## 1. Introduction

Alphaviruses (family: Togaviridae) are positive-sensed, single-stranded RNA and are related to arthropod-borne viruses (arboviruses) [1]. Arthritogenic alphaviruses are a group related to re-emergent “Old World” alphaviruses, globally distributed and genetically closed (Semliki Forest complex), including chikungunya virus (CHIKV), O’nyong-nyong virus (ONNV), Ross River virus (RRV), Barmah Forest virus (BFV), Mayaro virus (MAYV), Sindbis virus (SINV) and Semliki Forest virus (SFV), which have had recent outbreaks [2,3]. Arthritogenic alphavirus infection usually causes dengue-like symptoms (debilitating fever, rash and/or myalgia), but also severe chronic rheumatic disorders (polyarthralgia and polyarthritis) resulting from musculoskeletal inflammation [2,4]. 

**Figure 1:**
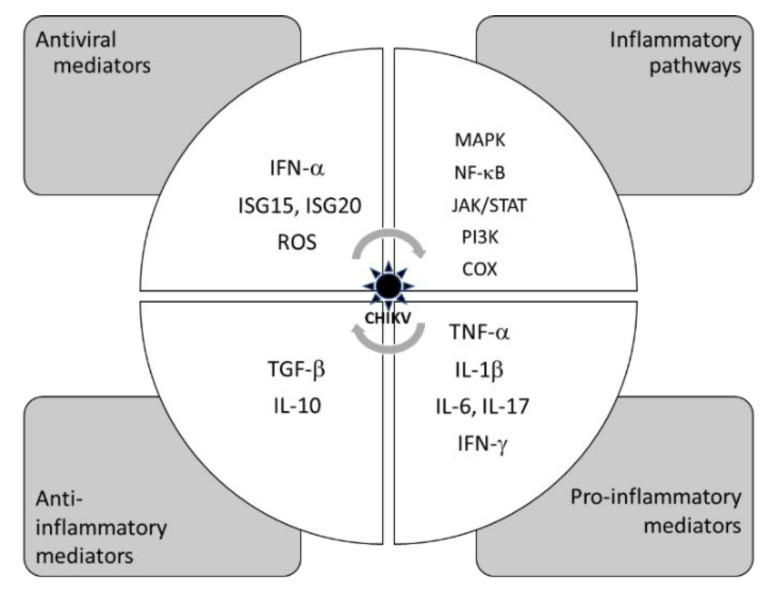
Simplified representation of antiviral and inflammatory components of the host immune system illustrated from cumulative experimental data for CHIKV infection and inflammation [5].

Recent knowledge on arthritogenic alphavirus infection highlighted the pivotal role of host immunity in protective antiviral and inflammatory responses but also an exuberant inflammatory response which can lead to the aforementioned severe complications [5]. As summarized in Figure 1 for the most investigated CHIKV, after viral detection and recognition, the complex immune response mainly involves the production of antiviral effectors (interferons (IFNs), interferon-stimulated genes (ISGs) and reactive oxygen species (ROS)), the release of pro-inflammatory cytokines through the activation of kinase signaling pathways (mitogen-activated protein kinase (MAPK), nuclear factor kappa B (NF-κB), Janus kinase/signal transducer and activator of transcription (JAK/STAT) or phosphoinositide 3-kinase (PI3K)) or the cyclooxygenase (COX)-dependent arachidonic cascade, and the subsequent attenuation of the inflammatory process via anti-inflammatory cytokines. The development of arthritic diseases was assumed on the viral infection of skeletal muscles and synovial tissues which upregulate pro-inflammatory cytokines (interleukin (IL)-6, tumor necrosis alpha (TNF-α) and IFN-β), as seen in patients infected with CHIKV [6], RRV [7] and MAYV [8]. Clinical data from CHIKV patients established that a robust cytokine profile is preventive to chronic joint pain during acute infection, while a low cytokine profile is predictive of chronic arthritis pathogenesis [9]. Thus, the regulation of the immune response is a key therapeutic strategy against arthritogenic alphavirus infection and induced inflammation. 

To date, there are no specific therapeutics for arthritogenic alphavirus infection and the related inflammatory diseases. For the recent investigations on CHIKV, viral inhibitors were mostly assessed using old drugs such as the broad-spectrum antiviral arbidol [10], the anti-IAV amantadine [11], the anti-trypanosomal suramin [12], the naturally occurring anti-cancer lobaric acid from the antarctic lichen *Stereocaulon alpinum* [13] and the antibiotic ribostamycin isolated from *Streptomyces ribosidificus* [14], or by designing novel synthetic series from triazolopyrimidinone [15] or thiazolidinone [16] scaffolds. Similarly, inhibitors of the essential host cell functions for the virus life cycle were evaluated with approved synthetic drugs, including the anti-malarial chloroquine (quinoline) [17], the antiparasitic atovaquone (naphthoquinone) [18] or the anti-depressant imipramine (dibenzoazepine) [19]. For the resulting inflammatory disorders, the available medications include non-steroidal anti-inflammatory drugs (NSAIDs) such as aspirin, ibuprofen or diclofenac used for the symptomatic relief of mild-to-moderate inflammation to decrease prostaglandin (PG) (e.g., PGE2) production via the inhibition of COX activity. Disease-modifying antirheumatic drugs (DMARDs), such as the well-investigated methotrexate (MTX) [20], are employed to alleviate chronic inflammation and decrease joint damage through the suppression of exuberant immune and/or inflammatory responses over the time, but without the relief of symptoms, as experienced in those with chronic CHIKV infection [21].

Immunomodulators are becoming an attractive therapeutic strategy for immune suppression and to decrease inflammation (immunosuppressants) or immune potentiation (immunostimulants) in cancer, cardiovascular, metabolic and immune-mediated inflammatory disorders or viral infections [22,23,24,25]. Immunomodulatory effects have been characterized for some antiviral compounds with prophylactic and/or therapeutic action against arthritogenic-alphavirus-induced damage, including (semi)-synthetic glycosaminoglycans mimetics such as the approved pentosane polysulfate used for the treatment of osteoarthritis [26] or the promising anticancer pixatimod previously investigated against RRV-induced cell damage [27,28], as well as tilorone (fluorenone) assessed for CHIKV [29] and earlier reported for SINV [30]. 

**Figure 2:**
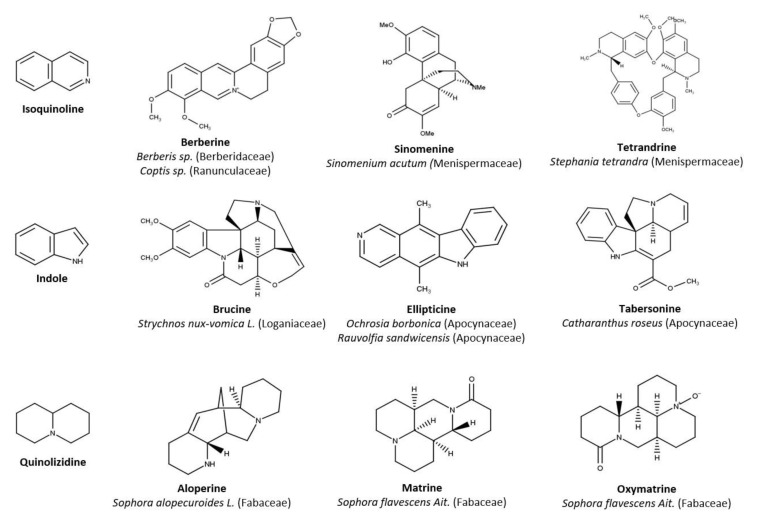
Chemical structures of the selected isoquinoline-, indole- and quinolizidine-derived alkaloids with their common botanical sources. Molecules were drawn using MarvinSketch.

Plant-derived immunomodulators have attracted increasing interest, with a large variety of chemical classes of natural compounds [31,32,33]. Alkaloids are a large and diverse group of nitrogen-containing compounds and are specialized or ‘secondary’ metabolites [34], mainly from plants of various botanical families, and exhibit a wide range of biological activities including antiviral and/or anti-inflammatory properties [35,36,37,38,39,40,41,42]. Nowadays, more than 27,000 alkaloids have been identified, but despite their drug-like features [43,44,45,46] and their traditional medicinal use, they are currently under-represented in marketed or licensed medicines (‘drugs’) [47]. Recently, we demonstrated the immunomodulatory effects in vitro of the quinoline-derived alkaloid camptothecin [48] against ONNV. Isoquinolines, indoles and quinolizidines are major chemical classes of biologically relevant alkaloids with potent antiviral activities [44,49,50] and anti-inflammatory effects [35]. The great potential of plant-containing alkaloids to modulate viral infections and related inflammation has been recently reviewed for prominent human pathogenic viruses, e.g., human immunodeficiency virus 1 (HIV-1), Influenza A virus (IAV) and hepatitis B or C viruses (HBV or HCV) [51]. Growing interest is being paid to the immunomodulatory activity of alkaloids [42], but their beneficial effects against arthritogenic alphaviruses have been poorly investigated. We discuss here the immunomodulatory effects of some alkaloids belonging to the isoquinoline, indole or quinolizidine classes from various botanical sources (Figure 2). These compounds were selected for their activities in vitro, ex vivo and/or in vivo established against various pathogenic viruses and chronic diseases. We highlighted the recent interest in alkaloids against arthritogenic alphavirus infection, as well as their immunomodulatory effects through the foremost mechanisms involved in arthritogenic alphavirus pathogenesis. 

## 2. Results and Discussion

In the recent challenge of drug discovery against alphaviruses, safe and efficient immunomodulators are expected to promote both a profitable antiviral immune response and a well-balanced inflammatory response. To date, there is little evidence of natural alkaloids that may serve as such a dual and attractive therapeutic strategy. Tomatidine, a natural steroidal alkaloid, has been recently reported as a valuable antiviral compound in vitro (EC_50_ = 1.3 µM; CC_50_ = 156 µM; SI 120) against CHIKV through the inhibition of the viral production, but additional immunomodulatory effects have not been established yet [52,53,54]. 

From the wide structural variety of alkaloids, we focused on three major classes based on their biological importance and their pharmaceutical relevance. Isoquinolines are the most structurally diverse and widely distributed group of alkaloids and are divided into 13 major subclasses derived from the benzylisoquinoline sub-unit represented here by berberine and sinomenine, or by a dimeric form (bisbenzylisoquinoline) with tetrandrine. The great value of isoquinoline alkaloids has been recently established for their multitarget potential in multifactorial chronic health conditions including immunological, metabolic and neurologic disorders [55]. Indole alkaloids are characterized by fused benzopyrrole ring systems which create large structural variety [56]. Brucine, ellipticine and tabersonine refer to the predominant monoterpene indole alkaloids which display various biological activities including anti-infectious and cytotoxic effects [57,58]. Quinolizidines include matrine- and aloperine-type alkaloids with multieffects evidenced by their anti-inflammatory, anti-tumoral, anti-microbial, cardioprotective or anti-atherosclerosis activity [59,60].

For the nine representative compounds, experimental data were selected for their dual antiviral and/or immunomodulatory effects in various health conditions (Table 1 and Table 2).

The antiviral activities of the reported alkaloids (Table 1) were characterized on both prominent RNA and DNA viruses, and their broad-spectrum activity is of interest to identify novel applications, as illustrated by the traditionally used and clinically promising isoquinoline berberine [61,62]; the latter proved to be efficient in disrupting the replication process of various DNA viruses [63] and has been also assessed against RNA viruses including CHIKV, SINV and SFV [64] or the novel severe acute respiratory syndrome related to Coronavirus-2 (SARS-CoV-2) [65]. These data support the broad-spectrum potential of berberine against genetically different viruses and at low micromolar range and low toxicity in vitro (EC_50_ = 1.8 µM; CC_50_ > 100 µM; SI > 127) for CHIKV [64] compared to its potency against the other reported RNA or DNA viruses. Of note, berberine demonstrated substantial inhibitory effects in vitro on the essential host component reverse transcriptase for HIV-1 (IC_50_ = 0.33 µM; CC_50_ = 2.09 µM; SI = 16.07) [66]. The inhibitory potential of the quinolizidines was not evidenced against arthritogenic alphaviruses but was supported with multiple targets on the virus life cycle for HCV attachment, replication and cell-to-cell transmission [67] or HIV-1 (fusion and entry) [68] using aloperine with satisfactory potency in vitro (EC_50_ = 7.06 µM; CC_50_ > 200 µM and EC_50_ = 1.75 µM; CC_50_ > 86.2 µM, respectively) and HBV replication in vivo using oxymatrine [69].

The above data established typical antiviral mechanisms related to viral clearance by interference on the virus life cycle (entry, replication, assembly and release) and/or the specific virus–host interactions (viral cell-to-cell transmission and enzyme activity such as reverse transcriptase for HIV-1) [79]. Interestingly, additional immunomodulatory effects of interest during viral infection were also evidenced. Such effects have been previously exploited for anti-HIV therapy [80] and more recently for the treatment of infection with SARS-CoV-2 viruses [81] or arthritogenic alphaviruses [2] to prevent the excessive release of cytokines that may cause inflammation or pathogenesis [25]. The selected experimental data in vitro and in vivo established the multifocal immunomodulatory effects of alkaloids during viral infection. 

First of all, the management of host antiviral signaling occurs via the downregulation of the pro-inflammatory inducer TNF-α, as shown in vitro for berberine [70] or oxymatrine [75] during IAV infection. Such an effect is of interest for the prominent role of TNF-α established during arthritogenic alphavirus infection. In contrast, alkaloids can increase the regulatory type II interferon IFN-γ as established with oxymatrine during HBV infection [69]. 

Secondly, the regulation of viral-related inflammation was demonstrated by the inhibition of inflammatory mediators or signaling pathways. The inhibition of PGE2 in vitro via berberine during IAV infection ascertained the sensitivity of the arachidonic cascade to alkaloids [70]. The downregulation of other inflammatory signaling pathways was supported in vitro via the inhibition of MAPK using berberine against RSV [71]. In vivo experiments demonstrated the multikinase effects (MAPK, NF-κB and PI3-AKt) of berberine against CHIKV [64] or oxymatrine against IAV [75]. Of note, the clinical value of matrine to resolve inflammation was recently supported by the reduction in C- reactive protein (CRP) levels against SARS-CoV-2 infection [73].

Finally, alkaloids could trigger an immune response during viral infection. As shown in vivo during HCV infection, oxymatrine increases the pro-inflammatory T_H_1-cytokines (IFN-γ and IL-2) for viral neutralization and decreases the regulatory T_H_2-cytokines (IL-4 and IL-10) [74]. Oxymatrine also increases IFN-γ against HBV in vivo (Sang et al. 2017). Remarkable immunosuppressive effects were shown in vitro using berberine against RSV [71] by the decreases in the crucial IL-6 in persistent arthralgia related to arthritogenic alphavirus infection [82], or in vitro and in vivo using oxymatrine against IAV [75] for the inhibition of IL-1β involved in the severity of arthritogenic alphavirus infection [83].

The immunomodulatory effects of the representative alkaloids were also demonstrated in various chronic health conditions not related to viral infection (Table 2). These effects are typically assessed at non-toxic doses of the tested compound.

**Table 2:** Immunomodulatory effects of alkaloids in non-viral related models.

Disease Model	Alkaloid	Model/Tested Doses	Effect	Ref.
Cancer	Berberine	In vitro, carcinoma cell (KB) and oral squamous carcinoma cell (OC2)/1, 10 or 100 μM In vivo, Wistar rats/1, 5 or 10 mg/kg	Inhibits PGE2 synthesis and COX-2 expression	[84]
		In vitro, human gastric cancer cell (SNU-5)/75 μM	Increases ROS production Inhibits NF-κB pathway	[85]
		In vitro, human thyroid carcinoma cell (C643, OCUT1, TPC1 and Htori3)/20 or 80 µM	Inhibits MAPK pathway	[86]
		In vitro, breast cancer cell (MDA-MB231 and MCF-7)/3.125–100 µM	Inhibits MAPK pathway	[87]
	Tetrandrine	In vitro, colon cancer cell (CT-26)/0.1–250 μM In vivo, tumor model mouse/50 and 150 mg/kg	Activates MAPK pathway for cell apoptosis	[88]
	Ellipticine	In vitro, human endometrial cancer cell (RL95-2)/ 0.1–10 μM	Increases ROS production and activates MAPK pathways for cell apoptosis	[89]
Cardiovascular disease	Matrine	In vivo, ischemia/reperfusion in rats/50 or 100 mg/kg Ex vivo, cardiomyocytes from rats/200 or 400 µM	Activates JAK/STAT pathway for a protective protein (HSP70)	[90]
Atherosclerosis	Aloperine	In vitro, ox-LDL induced inflammation in human umbilical vein endothelial cell (HUVEC)/50 and 100 µM	Decreases ROS production Inhibits pro-inflammatory IL-6 and MCP-1	[91]
Arthritis diseases	Berberine	In vivo, collagen-induced arthritis (CIA) in rats/200 mg/kg	Decreases pro-inflammatory TNF-α, IL-1β, IL-6 and IL-17 Inhibits MAPK pathway	[92]
		In vivo, Freund’s complete adjuvant (FCA) induced arthritis rats/75 and 150 mg/kg	Decreases pro-inflammatory IL-6 and IL-17 Increases the levels of regulatory IL-10 and TGF-β	[93]
		Ex vivo, Freund’s complete adjuvant (FCA) induced arthritic fibroblast-like synoviocytes (AA-FLS) from rats/15, 30 and 45 µM In vitro, bone-marrow-derived monocytes (BMMs)/15, 30 and 45 µM	Decreases pro-inflammatory TNF-α, IL-1β, IL-6 and IL-23 Inhibits PI3K pathway	[94]
	Oxymatrine	In vivo, collagen-induced arthritis (CIA) in rats—25, 50 and 100 mg/kg	Decreases pro-inflammatory TNF-α and IL-17A	[95]
		In vitro, rheumatoid arthritis fibroblast-like synoviocytes (RA-FLS)/10–100 μM	Decreases pro-inflammatory IL-6 and IL-8 Inhibits NF-κB and MAPK signaling pathways	[96]
Osteoarthritis	Berberine	In vitro, osteoarthritis synovial fibroblasts (OASFs)/5–100 µM	Inhibits pro-inflammatory IL-1β Inhibits NF-κB pathway	[97]
Arthritis diseases	Sinomenine	In vitro, rheumatoid arthritis fibroblast-like synoviocytes (RA-FLS)/0.1 and 1 μM	Decreases expression of proinflammatory TNF-α and IL-6 Inhibits COX and NF-κB pathways	[98]
		Ex vivo, fibroblast-like synoviocytes (FLS) of adjuvant-induced arthritis (AIA) rat/50–400 µM	Inhibits kinase (ERK/Egr-1) pathway	[99]
Chronic arthritis	Tetrandrine	In vivo, adjuvant-induced arthritis (AIA) rat/25 mg/kg/day or 80 mg/kg/day	Inhibits pro-inflammatory TNF-α and IL-1	[100]
Inflammation (other pathological conditions)	Brucine	In vivo, carrageenan-induced rat paw edema/15 and 30 mg/kg	Inhibits pro-inflammatory PGE2	[101]
	Ellipticine	In vitro, bone-marrow-derived macrophages (BMDMs)/5 μM In vivo, mouse with LPS-induced septic shock/20 mg/kg	Decrease pro-inflammatory TNF-α and IL-6 Inhibits NF-κB pathway	[102]
	Tabersonine	In vivo, LPS-induced inflammation in acute lung injury (ALI) mouse model/10, 20 or 40 mg/kg Ex vivo, bone-marrow-derived macrophages (BMDMs)/10 μM In vitro, human embryonic kidney cell (HEK293T)/10 μM	Inhibits pro-inflammatory TNF-α, IL-6 and IL-1β Inhibits NF-κB and MAPK pathways	[103]
	Oxymatrine	In vivo, Wistar rats after intracerebral hemorrhage (ICH)/120 mg/kg/day	Inhibits pro-inflammatory TNF-α, IL-1β and IL-6 Inhibits NF-κB pathway	[104]

Alkaloids proved to be efficient in resolving inflammation established as a risk factor and promoter of cancer, and they act through common pathways and biochemicals with inflammation induced by infection [105]. Defensive ROS production was promoted in several models of cancer with berberine [86] or ellipticine [89]. The downregulation of the arachidonic cascade was demonstrated by the decrease in PGE2 production and the inhibition of COX-2 at the transcriptional level using berberine [84]. Interference with the pivotal kinase inflammatory signaling pathways (MAPK, NF-κB and PI3-AKt) occurred with berberine [86,87], tetrandrine [88] and ellipticine [89]. Of note, the isoquinoline berberine demonstrated substantial immunomodulatory effects at low micromolar inhibitory concentrations in human thyroid carcinoma cell (IC_50_ = 0.891 μM) [86], to moderate concentrations in breast cancer cell (IC_50_ = 15 μM) [87], and at higher doses in human gastric cancer cell (IC_50_ = 48 μM) [85]. Apoptosis in colon cancer (SNU-5) cells using the isoquinoline tetrandrine [88] and in human endometrial cancer (RL 95-2) cells treated with the indole ellipticine [89] underlined an essential antiproliferative effect of these alkaloids [106]. 

The representative matrine and aloperine quinolizidines showed multiple and valuable immunomodulatory effects in complex pathologies. In a cardiovascular model, matrine demonstrated the in vivo and ex vivo activation of the JAK2/STAT3 signaling pathway [90] with the upregulation of the HSP70 protein which had a cardioprotective effect [107]. In atherosclerosis, aloperine demonstrated a protective effect in vitro via the downregulation of the key immune effectors including excessive ROS, the pro-inflammatory cytokine IL-6, the signaling chemokine MCP-1 and the inducible endothelial cell adhesion molecules, vascular cell adhesion molecule 1 (VCAM-1) and E-selectin. 

Rheumatoid arthritis is a chronic, systemic autoimmune and inflammatory disease which causes damage to joint tissues, and alphavirus infection results in rheumatoid-arthritis-like symptoms and complications. The immunomodulatory effects of alkaloids in the reported arthritic disorders were supported by robust experimental models: in vitro using bone-marrow-derived monocyte macrophages (BMMs) [108] or osteoarthritis synovial fibroblasts (OASF) [109]; ex vivo using key effectors rheumatoid arthritis fibroblast-like synoviocytes (RA-FLS) [110]; and in vivo using inflammatory stimulators in preclinical test models via adjuvant-induced arthritis (AIA) in rats including collagen-induced arthritis (CIA) [111] or Freund’s complete adjuvant (FCA)-induced arthritis [112]. As illustrated for the overall arthritic diseases with the isoquinolines berberine [92,93,94], sinomenine [98,99] and tetrandrine [100] and the quinolizidine oxymatrine [95,96], the suppression of inflammatory signaling pathways decreases the common pro-inflammatory cytokines (e.g., TNF-α, IL-1β and IL-6), as well as the critical IL-17 involved in inflammation and bone loss in rheumatoid arthritis and also evidenced in CHIKV chronically affected patients [82]. 

The immunomodulatory effects of alkaloids were assessed on other inflammation test models in vitro, ex vivo or in vivo, as illustrated with the isoquinoline tabersonine [103], the indole alkaloid brucine [101] and ellipticine [102] and the quinolizidine oxymatrine [104]. The data highlighted similar effects to those previously discussed, and noticeably, the suppression of kinase signaling pathways (MAPK and NF-κB) and the downregulation of pro-inflammatory mediators (TNF-α, IL-6, IL-1β and PGE2) which are critical during arthritogenic alphavirus infection and related inflammation.

**Figure 3:**
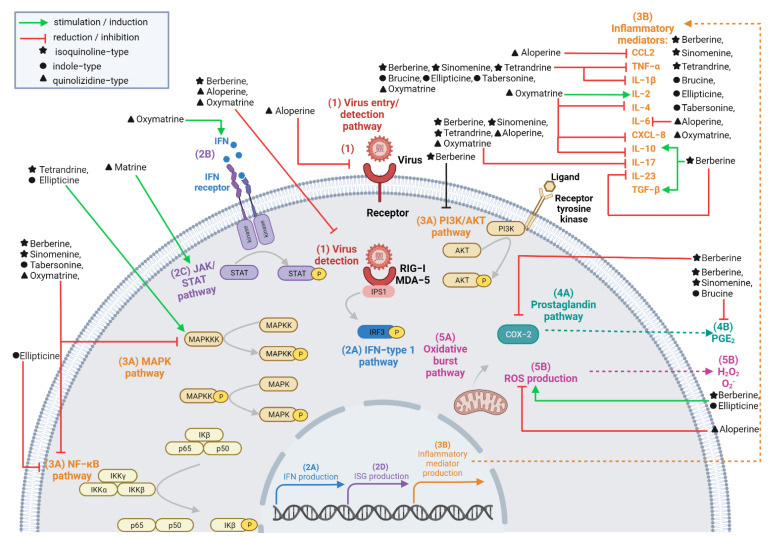
Effects of the selected alkaloids on virus detection (1), antiviral and/or inflammatory signaling pathways (2A), (2C), (3A), (4A) or (5A) and mediators (2A), (2B), (2D), (3B), (4B) and (5B). The figure was created using biorender.com.

As summarized in Figure 3 from the reported data (Table 1 and Table 2), the selected alkaloids have substantial effects on various host-based targets involved in viral infection and inflammation. Antiviral immune response is mediated by cell-surface (receptor) or cytosolic viral sensors (e.g., RIG-I and MDA-5 for alphaviruses) involved in viral entry or detection (1). The inhibition of these host cell components, as evidenced with the isoquinoline berberine and the quinolizidines oxymatrine and aloperine, prevents viral invasion. Alternatively, the quinolizidine oxymatrine proved to be able to mount an antiviral response mediated by IFN production (2A) and signaling via the IFN cell-surface receptor (2B). As shown, the selected alkaloids, especially the isoquinoline berberine, can interfere with different inflammatory signaling pathways, including the JAK/STAT pathway (2C), the kinase pathways (3A), the COX-2-related pathways (4A) and (4B), as well as the oxidative pathways (5A) and (5B). The inhibitory and/or inductive effects may be of interest to achieve a well-balanced inflammatory response and consequently, to prevent complications resulting from the dysregulation of the immune system. The potential of the selected alkaloids was also established in the upregulation and/or downregulation of the major inflammatory mediators.

The combined data support the immunomodulatory effects of the selected alkaloids against arthritogenic alphaviruses as the latter mobilizes the same host key functions. 

Plant-derived alkaloids are major sources for drug discovery, with highly potent naturally occurring compounds, including isoquinolines (e.g., the anticancer noscapine and the analgesics morphine and codeine), indoles (e.g., the anticancer vinblastine and vincristine) and quinolizidines (e.g., the anti-Influenza A oseltamivir and zanamivir) [43,113], and they are gaining increasing consideration in managing viral infection and inflammation [51]. However, the pharmaceutical development of natural alkaloids is often faced with the problem of their low activity and/or their toxic effects [114]. Therefore, many efforts are devoted to the structural optimization of an original alkaloid and to identifying ‘druggable’ derivatives. As exemplified in some of the reported studies, structure-activity relationship (SAR) studies have been successfully applied to improve potent viral inhibitory effects. In vitro anti-HIV assays on a set of *O*-esters derivatives of the berberine allowed two compounds with better therapeutic indexes for antiviral activity to be identified [66], in line with intensive efforts to promote benzylisoquinoline alkaloids [44]. The valuable quinolizidine aloperine and N-substituted derivatives have been established as a new class of HIV entry inhibitors [68]. The most potent molecules against alphaviruses are so far limited [115], and there is no evidence for structural optimization directed to the discovery of efficient immunomodulators of both antiviral and inflammatory responses.

## 3. Conclusions

From robust experimental models of viral infection and/or inflammation, the selected isoquinoline-, indole- and quinolizidine-derived alkaloids demonstrated substantial immunomodulatory effects. As evidenced, they can interfere with the canonical signaling pathways and biochemicals of the immune system. The effects of these alkaloids on arthritogenic alphavirus infection and related inflammation have been poorly studied, and the collective data ascertain the great potential of natural alkaloids or more toxicologically acceptable derivatives from structure–activity relationship studies. The most convincing evidence arises from the isoquinoline berberine, which can disrupt not only CHIKV replication but also the resulting immunological disorders. Other compounds investigated concerning other viruses or diseases shed light on the diverse mechanisms of action of interest for their application as immunomodulators against arthritogenic alphavirus infection and related inflammation.

## Figures and Tables

**Table 1:** Antiviral and immunomodulatory effects of alkaloids during viral infection.

Virus	Alkaloid	Model/Tested Doses	Efficacy and/or Safety In Vitro ^(a)^	Effect	Ref.
**Viral RNA**					
Influenza A virus (IAV)	Berberine	In vitro, murine macrophage-like cell (RAW 264.7)/25 μM	IC_50_ = 0.01 μM	Inhibits virus growth Decreases pro-inflammatory TNF-α and PGE_2_	[70]
Respiratory syncytial virus (RSV)		In vitro, human adenocarcinoma alveolar basal epithelial cell (A549)/25 or 100 µM	CC_50_ > 100 μM	Inhibits pro-inflammatory IL-6	[71]
Chikungunya virus (CHIKV)		In vitro, human osteosarcoma cell (HOS)/0.1–800 μM In vivo, mouse/10 mg/kg	EC_50_ = 12.2 μM CC_50_ = 429.5 μM SI = 35	Inhibits MAPK/PI3K-AKt signaling pathways Reduces viral load and joint swelling in mice	[72]
Chikungunya virus (CHIKV), Semliki Forest virus (SFV) and Sindbis virus (SINV)		In vitro, baby hamster kidney cell (BHK)/3 µM	For CHIKV: EC_50_ = 1.8 μM CC_50_ > 100 μM SI > 27	Inhibits viral replication	[64]
Severe acute respiratory syndrome related to Coronavirus-2 (SARS-CoV-2)		In vitro, primary human nasal epithelial cell/10 μM	EC_50_ = 9.1 μM CC_50_ > 150 μM SI > 16	Inhibits viral replication	[65]
Severe acute respiratory syndrome related to Coronavirus-2 (SARS-CoV-2)	Matrine	In vivo, human	-	Decreases sera levels of lymphocytes and CRP	[73]
Hepatitis B virus (HBV)	Oxymatrine	In vivo, mouse/200 mg/kg/day	-	Increases pro-inflammatory T_H_1-cytokines (IFN-γ and IL-2) Decreases anti-inflammatory T_H_2-cytokines (IL-4 and IL-10)	[74]
Influenza A virus (IAV)		In vitro, human adenocarcinoma alveolar basal epithelial cell (A549)/190 μM In vivo, mouse/60 or 120 mg/kg	EC_50_ = 64.47 μM AI = 3868.1359	Decreases pro-inflammatory IL-1β, IL-6, IL-8 and TNF-α Inhibits MAPK and NF-κB pathways	[75]
Hepatitis C virus (HCV)	Aloperine	In vitro, human-derived hepatoma cell (Huh 7.5)/20 μM	EC_50_ = 7.06 μM CC_50_ > 200 μM	Inhibits virus attachment and replication Blocks viral cell-to-cell transmission	[67]
**Viral DNA**					
Herpes virus simplex 1 and 2 (HSV-1 and HSV-2)	Berberine	In vitro, Vero cells/2400 μM	HSV-1: IC_50_ = 24.4 μM CC_50_ = 39.2 μM SI = 161 HSV-2: IC_50_ = 26.7 μM CC_50_ = 39.2 μM SI = 141	Inhibits viral replication and DNA synthesis	[76]
Human immunodeficiency virus 1 (HIV-1)		In vitro, T cells containing a plasmid encoding the green fluorescent protein (CEM-GFP)/0.29 μM	IC_50_ = 0.33 μM CC_50_ = 2.09 μM SI = 16.07	Inhibits reverse transcriptase (RTase) activity	[66]
Human cytomegalovirus (HCMV)		In vitro, human foreskin fibroblasts (HFF)/2.65 ± 0.35 µM	EC_50_ = 2.65 μM CC_50_ = 390 μM SI = 147	Inhibits viral E protein gene expression	[77]
Hepatitis B virus (HBV)	Oxymatrine	In vivo, mouse/200 mg/kg	-	Decreases antigen expression	[78]
Hepatitis B virus (HBV)		In vivo, mouse/20.0 mg/kg	-	Inhibits viral replication Decreases antigen levels Promotes IFN-γ	[69]
Human immunodeficiency virus 1 (HIV-1)	Aloperine	In vitro, fibroblast-like COS cells and TZM-bl cells—0.01–10 μM	EC_50_ = 1.75 μM CC_50_ > 86.2 μM	Inhibits virus fusion and entry	[68]

^(a)^ IC_50_: 50% inhibitory effect; EC_50_: 50% cytotoxic concentration; CC_50_ = 50% cytotoxic concentration; SI: selectivity index; AI: antiviral index.

## Data Availability

On demand–EPI research unit.
